# History of Medicine

**Published:** 1903-02

**Authors:** 


### Books and Magazines.

---

**All books intended for review in the Texas Medical Journal must be sent to Associate Editor Dr. W. B. Russ, San Antonio, Texas.**

---

History of Medicine.—A brief outline of medical history and sects of physicians, from the earliest historic period; with an extended account of the new schools of the healing art in the nineteenth century, and especially a history of the American eclectic practice of medicine, never before published. By Alexander Wilder, M. D., Newark, N. J., Honorary Member of the Liverpool (Eng.) Anthropological Society, Vice-President of the "American Akademe," etc. First thousand. Pp. 946. Price, $2.75. New Sharon, Maine: New England Eclectic Publishing Co. 1901.

The author in the outset seems to regret that "A one catholic science of medicine, of inerrant orthodoxy and faultlessly classified, cannot be intelligently affirmed to exist." Still the chief purpose of the work, as shown throughout its pages, is to defend not only eclecticism but sectarianisms in general. The author fails to name any great scientific achievements that have been accomplished by sectarian medicine. True science is not bounded by hobbies, which are but narrowing freaks of the human mind at least. As a good and faithful record of the author's side of the question, it is doubtful whether there is any other book half as valuable to its friends as the present volume. T. J. B.

